# Association of Urine Albumin/Creatinine Ratio below 30 mg/g and Left Ventricular Hypertrophy in Patients with Type 2 Diabetes

**DOI:** 10.1155/2020/5240153

**Published:** 2020-01-21

**Authors:** Xiangkun Xie, Zhengliang Peng, Hanlin Li, Dan Li, Yan Tu, Yujia Bai, Xingfu Huang, Wenyan Lai, Qiong Zhan, Qingchun Zeng, Dingli Xu

**Affiliations:** ^1^State Key Laboratory of Organ Failure Research, Department of Cardiology, Nanfang Hospital, Southern Medical University, Guangzhou, China; ^2^Department of Cardiology, Sun Yat-sen Memorial Hospital, Sun Yat-sen University, Guangzhou, China; ^3^Laboratory of Cardiac Electrophysiology and Arrhythmia in Guangdong Province, Guangzhou, China; ^4^EICU, The First Affiliated Hospital of University of South China, Hengyang, Hunan, China; ^5^Division of Nephrology, Nanfang Hospital, Southern Medical University, Guangzhou, China

## Abstract

Several studies show that even a level of urine albumin/creatinine ratio (UACR) within the normal range (below 30 mg/g) increases the risk of cardiovascular diseases. We speculate that mildly increased UACR is related to left ventricular hypertrophy (LVH) in patients with type 2 diabetes mellitus (T2DM). In this retrospective study, 317 patients with diabetes with normal UACR, of whom 62 had LVH, were included. The associations between UACR and laboratory indicators, as well as LVH, were examined using multivariate linear regression and logistic regression, respectively. The diagnostic efficiency and the optimal cutoff point of UACR for LVH were evaluated using the area under the receiver operating characteristic curve (AUC) and Youden index. Our results showed that patients with LVH had significantly higher UACR than those without LVH (*P* < 0.001). The prevalence of LVH presented an upward trend with the elevation of UACR. UACR was independently and positively associated with hemoglobin A1c (*P* < 0.001). UACR can differentiate LVH (AUC = 0.682, 95% CI (0.602–0.760), *P* < 0.001). The optimal cutoff point determined with the Youden index was UACR = 10.2 mg/g. When categorized by this cutoff point, the odds ratio (OR) for LVH in patients in the higher UACR group (10.2–30 mg/g) was 3.104 (95% CI: 1.557–6.188, *P*=0.001) compared with patients in the lower UACR group (<10.2 mg/g). When UACR was analyzed as a continuous variable, every double of increased UACR, the OR for LVH was 1.511 (95% CI: 1.047–2.180, *P*=0.028). Overall, UACR below 30 mg/g is associated with LVH in patients with T2DM. The optimal cutoff value of UACR for identifying LVH in diabetes is 10 mg/g.

## 1. Introduction

Left ventricular hypertrophy (LVH) is an important factor in the occurrence of cardiac remodeling and cardiovascular events [1]. Diabetes is a risk factor of LVH, which is independent of hypertension [2]. Because of the high prevalence of hypertension in diabetes and its effect on cardiac structure independently of primary diseases [3], LVH has a high prevalence in diabetes. A previous study reported that about 70% of patients with type 2 diabetes mellitus (T2DM) had LVH [4]. It is even a stronger predictor of cardiovascular events than triple vessel coronary disease [5, 6]. One possible reason is that LVH is an early event in the development of arrhythmia, diastolic heart failure, ischemia, and atrial fibrillation [7]. Both electrocardiograph and echocardiography can be used in LVH diagnosis, but the former has a low sensitivity and it is expensive to screen for LVH in all patients with diabetes with the latter. In China, with the increasing prevalence of T2DM from 0.67% in 1980 to 10.4% in 2013 [8], it would be unrealistic to perform echocardiographic examination for all T2DM. Therefore, it is necessary to identify a sensitive and simple enough marker for LVH in T2DM, which can be used for risk stratification, especially in primary care.

Several mechanisms such as endothelial dysfunction, insulin resistance, and accelerated accumulation of advanced glycation end products (AGEs) have been proposed in order to explain the existing relationship between diabetes and LVH. The Steno hypothesis believes that albuminuria reflects widespread endothelial dysfunction, not only renal function impairment [9]. It has been reported that certain urinary proteins play a significant role in other systemic diseases, not just in urogenital diseases [10–12]. Urinary albumin/creatinine ratio (UACR) in a random spot urine is the easiest method to screen for albuminuria. Normal UACR is generally defined as <30 mg/g, used for kidney damage screening in patients with diabetes [13]. Despite robust evidence of the relationship between abnormal UACR and LVH in patients with diabetes, it is known that even a level of UACR below 30 mg/g increases the risk of cardiovascular diseases (CVDs). Recent studies have demonstrated that normal UACR is associated with an elevated risk of CVD mortality and incident hypertension, but not incident diabetes, which indicated that a higher level of UACR might be provoked by endothelial dysfunction, rather than a causal factor [14].

Based on these findings, we speculate that even if UACR is in the normal range, an altered level of UACR is associated with LVH prevalence. Therefore, we conduct a cross-sectional study to investigate the relationship between UACR below 30 mg/g and LVH in patients with diabetes.

## 2. Materials and Methods

### 2.1. Study Population

This is a retrospective study. Records of consecutively admitted patients between June 2016 and June 2018 to Nanfang Hospital, Southern Medical University, for evaluation or treatment of T2DM were reviewed. Patients with any of following conditions were excluded: (1) <18 years old; (2) congenital heart disease or primary pulmonary arterial hypertension; (3) urinary albumin/creatinine ratio (UACR) ≥30 mg/g; (4) a history of known coronary heart disease, coronary artery bypass or angioplasty, and severe valvular heart disease; and (5) concurring pregnancy or infection. For those with several hospitalizations, only records from the first hospitalization were included. The study was approved by the institutional review board of Nanfang Hospital. No informed consent was required because the data in our study were anonymized.

### 2.2. Data Collection

All demographic characteristics were obtained from electronic medical records of Nanfang Hospital including age, gender, weight, height, body mass index (BMI), smoking, duration of diabetes, medication history, systolic blood pressure (SBP), diastolic blood index (DBP), and comorbidities. The blood samples were taken after fasting for 12 h overnight, and the first morning urine samples were collected within 24 h of admission. Hemoglobin (HGB), hematocrit (HCT), total cholesterol (CHOL), low-density lipoprotein cholesterol (LDL-c), high-density lipoprotein cholesterol (HDL-c), triglyceride (TG), fasting plasma glucose (FPG), hemoglobin A1c (HbA1c), fasting insulin (FINS), albumin (ALB), serum creatinine (Cr), urine acid (UA), urea nitrogen (UREA), phosphate, sodium, and chlorine were all collected from hospital database. Urine albumin was measured by immunoturbidimetric assay, and urinary creatinine concentration was measured by enzymatic method. As recommended by the American Society of Echocardiography [15], transthoracic echocardiography was performed by a senior echocardiographer. Color Doppler ultrasonic diagnostic apparatus by German Siemens Company (Siemens Sequoia 512 Encompass) was used for the examination, with patients in partial left lateral decubitus position. Bilateral carotid ultrasonography was performed according to standards for carotid ultrasound examination in Chinese healthy population [16].

### 2.3. Definition of Covariates

Anemia was defined according to the Chinese Society of Hematology expert consensus on iron deficiency anemia as HGB < 120 g/L for men and HGB < 110 g/L for women [17]. Obesity was defined as BMI ≥ 28 kg/m^2^ according to the Chinese standard [18]. Smoking was defined as “ever smoked.” The estimated glomerular filtration rate (eGFR) was calculated using the Modification of Diet in Renal Disease (MDRD) equation [19]. Insulin resistance was estimated by the homeostatic model: HOMA-IR = FPG (mmol/L) × FINS (mIU/L)/22.5 [20]. According to the European Association of Cardiovascular Imaging and the American Society of Echocardiography [21], relative wall thickness (RWT) was calculated as the ratio of two times posterior wall thickness to end-diastolic left ventricular (LV) diameter and increased RWT was defined as >0.42. Left ventricular mass (LVM) was estimated according to the formula: LVM (*g*) = 0.8 × 1.04 × [(LVIDd (cm) + LVPWd + IVSd)^3^ − LVIDd^3^] + 0.6. Normalization of LVM for height to the power of 2.7 was regarded as the left ventricular mass index (LVMI). LVH was defined as follows: LVMI > 48 g/m^2.7^ for men and LVMI > 44 g/m^2.7^ for women. LV geometry was defined as normal (normal LVMI and normal RWT); concentric remodeling (normal LVMI and increased RWT); eccentric hypertrophy (increased LVMI and normal RWT); and concentric hypertrophy (increased LVMI and increased RWT).

### 2.4. Statistical Analysis

Normally distributed continuous variables were expressed as the mean value ± standard deviation, while nonnormally distributed variables were expressed as the median with interquartile ranges. Differences in normally distributed variables were determined by independent-sample *T* test or one-way ANOVA or Kruskal–Wallis tests. Homogeneity of variance was explored by the Levene test, and a *P* value less than 0.1 was considered heterogeneity of variance. Nonparametric test was used for comparing the difference of nonnormally distributed variables. Categorical variables were reported as numbers and percentages, and chi-square test was used for comparing proportions. Multivariable linear regression analysis was applied to explore an independent association between UACR and other clinical parameters. UACR was logarithmically transformed to approximate normal distribution. The ability to differentiate LVH of UACR was evaluated using the area under the curve (AUC) in the receiver operating characteristic (ROC) curve. Multivariable logistic regression analysis was used for determining the variables associated with LVH and identifying the association between LVH and UACR. A two-sided *P* value of <0.05 was considered statistically significant. All analyses were performed using SPSS version 22.0 (IBM SPSS Statistics, IBM Corporation, Armonk, New York).

## 3. Results

### 3.1. Clinical Characteristics

**Figure 1:**
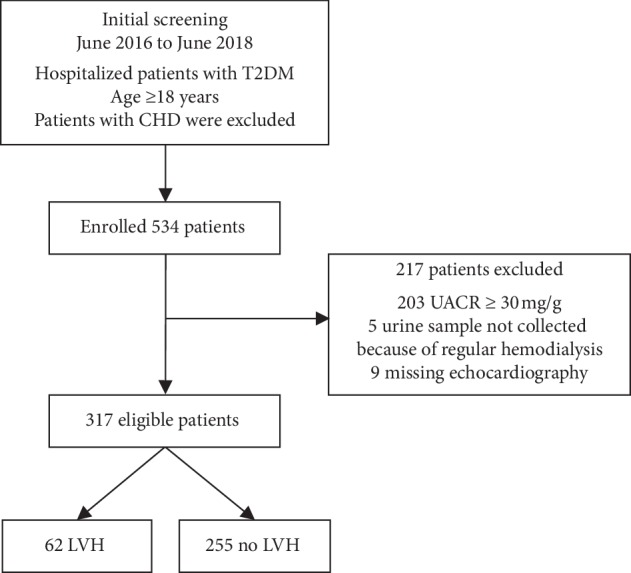
Inclusion flowchart for the study. T2DM: type 2 diabetes mellitus; CHD: coronary heart disease; UACR: urine albumin/creatinine ratio; LVH: left ventricular hypertrophy.

In our study, 534 patients were enrolled, and after exclusion, a total of 317 patients were included in the statistical analysis (Figure 1); 39.4% were female, with a mean age of 55.2 ± 12.1 years. The median duration of diabetes was 6 (1–10) years. LVH, hypertension, carotid plaque, atrial fibrillation (AF), obesity, and anemia were presented in 62 (19.6%), 119 (37.5%), 50 (15.8%), 3 (0.9%), 47 (14.8%), and 29 (9.1%) patients, respectively. Patients' characteristics in subjects with non-LVH and in those with LVH are shown in Table 1.

Significant difference in UACR was observed between non-LVH and LVH groups (6.2 (4.4–10.6) vs. 11.5 (6.0–21.2) mg/g, *P* < 0.001). Patients with LVH tended to have higher percentage of the subjects with hypertension, obesity, and the use of angiotensin-converting enzyme inhibitor/angiotensin receptor blocker (ACEI/ARB) and calcium channel blocker (CCB), be older, and fewer were males. Laboratory values such as HbA1c, FPG, HOMA-IR, and eGFR did not demonstrate any statistically significant difference between the two groups.

Patients' characteristics in subjects categorized by UACR quartiles are listed in Table 2. From UACR quartile 1 to quartile 4, the prevalence of LVH significantly rose from 10.8% to 36.7% (*P* < 0.001). There was a significant increase in the usage rate of ACEI/ARB and HbA1c and a significant decrease in ALB across UACR quartiles. Nevertheless, there was a remarkably similar usage rate of CCB and eGFR across UACR quartiles.

### 3.2. Association with the UACR Levels

We performed single regression and multiple regression analysis between HbA1c, ALB, age, gender, hypertension, smoking, the use of ACEI or ARB medication, and log_2_ UACR levels (Table 3). There was a significantly negative correlation between the log_2_ UACR levels and ALB (*P*=0.003), while significantly positive correlation was observed between log_2_ UACR and HbA1c (*P* < 0.001), independent of hypertension, the use of ACEI or ARB medication, smoking, age, and gender.

### 3.3. Association with LVH

To investigate the variables associated with LVH, we performed backward stepwise multinomial logistic regression analysis to include gender, age, hypertension, duration of diabetes, smoking, ALB, obesity, SBP, HDL-c, eGFR, log_2_ HOMA-IR, carotid plaque, log_2_ UACR, the use of ACEI or ARB, CCB, and statin medication on first step, which indicated that LVH was independently associated with gender, age, hypertension, obesity, and log_2_ UACR (Table 4).

**Figure 2:**
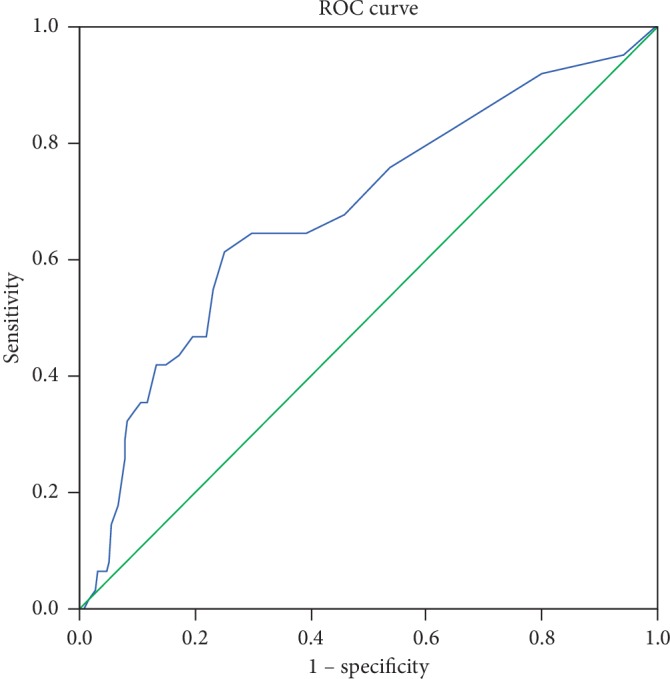
ROC curves of the ability of UACR to differentiate left ventricular hypertrophy.

The receiver operating characteristic (ROC) curve to differentiate patients with LVH yielded an area under the curve (AUC) of 0.682 (95% CI: 0.602–0.760, *P* < 0.001) for UACR (Figure 2). An optimal cutoff value for UACR of 10.2 mg/g for LVH was determined with the Youden index. Sensitivity, specificity, positive predictive value, and negative predictive value, respectively, were 61.3%, 74.9%, 75.2%, and 88.8%.

The ORs (95% CI) for LVH according to changes in UACR levels were shown by logistic regression analysis when UACR is a categorical variable (an optimal cutoff value according to the maximum Youden index) or a continuous variable (log_2_ UACR) (Table 5). Compared to UACR < 10.2 mg/g group, the OR for LVH was 3.104 (95% CI: 1.557–6.188, *P*=0.001) in UACR > 10.2 mg/g group, after adjustment for age, gender, and obesity in model 1, further adjustment for hypertension, carotid plaque, and duration of diabetes in model 2, and furthermore adjustment for smoking, the use of ACEI/ARB, and statin medication in model 3. As a continuous variable, for every increase of 100 percent in UACR level, the OR for LVH was 1.511 (95% CI: 1.047–2.180, *P*=0.028) in the fully adjusted model.

## 4. Discussion

We report here a cross-sectional study to investigate the association of UACR below 30 mg/g and left ventricular hypertrophy in patients with type 2 diabetes in South China. We found that even if UACR was in the normal range, a high UACR level was significantly associated with the prevalence of LVH, which was independent with the effect of age, gender, obesity, hypertension, carotid plaque, duration of diabetes, smoking, the use of ACEI/ARB, and statin medication.

In fact, there are many studies demonstrating that a high level of UACR is associated with an increased risk of cardiovascular diseases such as ischemic electrocardiographic abnormalities [22], coronary artery calcification score, and carotid intima-media thickness [23] in nondiabetic patients or diabetic patients. LIFE study discovered that increased UACR contributed to increasing risk of cardiovascular events without thresholds or plateaus [24]. In 2013, Gutiérrea et al reported that the effect of UACR on cardiovascular outcomes differed by race, with nonwhite being more susceptible than whites [25]. In 2017, Siddique et al. demonstrated that mildly increased UACR (10 mg/g−30 mg/g) was associated with a 1.4 times increase in all-cause mortality (*P*=0.042) in 2176 patients with diabetes with coronary heart disease by a post hoc analysis of BARL-2D study [26]. However, they did not investigate the relationships between the changes of heart structure and a mildly elevated UACR (<30 mg/g) in T2DM. We cannot know the reason why mildly elevated UACR increases the risk of cardiovascular events.

Previous studies have demonstrated an absolute association between T2DM and LVH [2, 27, 28]. T2DM is a significant trigger for endothelium damage, myocardial infarction, and heart failure. Traditional factor such as blood pressure can only explain about 25% of the variability in left ventricular mass [29]. ACEI/ARB use can reduce the risk of LVH but not cure it [4]. On the other hand, LVH is one of the clinical manifestations of diabetic cardiomyopathy [30, 31]. Considering the relationships between these diseases, it is significantly important to make an early diagnosis of LVH in T2DM.

In this study, we find that existing LVH may be invoked to explain the relationships between a mildly elevated UACR (<30 mg/g) and the risk of cardiovascular events. Our results suggested that 10 mg/g is the optimal cutoff points for identifying LVH in T2DM. Although Somaratne et al. also investigated the value of serum NT-proBNP to screen for LVH in T2DM, the results showed that serum NT-proBNP was unsuitable for a screening tool because of the influence of obesity or other metabolic risk factors [32]. The detection of UACR is less burdensome than timed or 24 h collections, bringing patients a lot of convenience. Measurement of UACR level has been general for T2DM, but UACR below 30 mg/g is often ignored. As we know, based on screening for renal damage, normal UACR is generally defined as <30 mg/g. It is more appropriate if <10 mg/g can be defined as a normal range of UACR to screen for left ventricular remodeling. Previous studies have shown that hypertension is often accompanied in patients with T2DM and can cause left ventricular diastolic dysfunction. Early echocardiography is recommended for diagnosing left ventricular diastolic dysfunction [33]. For the high prevalence of T2DM, UACR may also become a cheaper method for primary assessment of left ventricular structure and function. Moreover, we report that HbA1c level had a positive correlation with UACR, independent of hypertension, even in T2DM patients with a normal range of UACR, which suggested that there are some mechanisms between glycemic control and urinary albumin excretion. In our study, we did not find a significant difference between HOMA-IR and the prevalence of LVH. Previous studies have shown the considerable effect of brain natriuretic peptide (BNP) in counteracting IR. BNP can improve IR by reducing BMI via fat oxidation [34] or triglyceride lipolysis [35]. Serum NT-proBNP levels in LVH are higher than those in non-LVH [32], which may account for our results.

As we all know, UACR and eGFR are both biomarkers of kidney disease and both associate with systematic endothelial dysfunction. However, in our study, there is no significant difference in eGFR between the high UACR group and the low UACR group. The possible cause is that eGFR level estimated by the MDRD equation is not accurate enough, or UACR level is more sensitive for detecting subclinical renal damage than eGFR level. If these results are confirmed in further studies, UACR levels and its novel cutoff value can prove to be an economical and practical method for initial screening and follow-up of LVH in T2DM. Patients with T2DM who have more than 10 mg/g of UACR can be recommended to undergo echocardiography by experienced professionals. Since some research studies about the treatment of heart failure attach importance to the alleviation of LVH [36], it can be expected that the timely screening of LVH in T2DM will be paid more and more attention.

## 5. Limitations

Our study had several limitations. Firstly, this is a retrospective study and the results need validation in a prospective trial. For the retrospective nature of this study, we could not get the data of repeated measurement of echocardiographic parameters. But in our study, all patients' echocardiography was performed by the same senior echocardiographer, so the results were reliable and stable. Secondly, UACR level can change from day to day, but we only measured UACR a single time, which might result in inaccuracy of measurement. However, there are a number of evidences indicating that a single-voided test is reliable in screening for diseases [37, 38]. Finally, the insulin resistance index calculated by the homeostatic model in patients with diabetes was less accurate than in patients without diabetes, so the results were for preliminary reference only.

## 6. Conclusion

In summary, this study found that a high level of UACR is associated with LVH in T2DM. The optimal cutoff value for screening for LVH in T2DM is 10 mg/g. Further investigation is necessary to better manage diabetes with mildly increased UACR (10–30 mg/g).

## Figures and Tables

**Table 1:** Characteristics of patients grouped by left ventricular hypertrophy.

Variables	LVH		*P* value
	No (*n* = 255)	Yes (*n* = 62)	
Age (years)	53.3 ± 11.8	63.0 ± 10.5	<0.001
Female, *n* (%)	80 (31.4)	45 (72.6)	<0.001
Smoking, *n* (%)	71 (27.8)	6 (9.7)	0.003
Hypertension, *n* (%)	75 (29.4)	44 (71.0)	<0.001
Carotid plaque, *n* (%)	39 (15.3)	11 (17.7)	0.635
Duration of diabetes (years)	5 (1–10)	9 (5–13)	0.001
SBP (mmHg)	132.3 ± 17.3	139.6 ± 19.6	0.004
DBP (mmHg)	81.1 ± 10.3	80.9 ± 12.3	0.925
BMI (kg/m^2^)	24.3 ± 3.6	25.9 ± 5.5	0.025
Obesity, *n* (%)	30 (11.8)	17 (27.4)	0.002

*Medication history*			
ACEI/ARB use, *n* (%)	42 (16.5)	27 (43.5)	<0.001
Statin use, *n* (%)	102 (40.0)	30 (48.4)	0.230
CCB use, *n* (%)	24 (9.4)	17 (27.4)	<0.001
*β*-Blocker, *n* (%)	11 (4.3)	5 (8.1)	0.375

*Laboratory values*			
HbA1c (%)	8.5 (6.9–10.4)	8.9 (6.6–10.6)	0.948
HOMA-IR	1.45 (0.71–2.81)	1.51 (0.81–3.66)	0.457
FPG (mmol/L)	6.9 (5.4–8.6)	6.9 (5.8–8.6)	0.765
Anemia, *n* (%)	21 (8.2)	8 (12.9)	0.253
CHOL (mmol/L)	4.80 ± 1.03	4.85 ± 1.16	0.755
LDL-c (mmol/L)	3.03 ± 0.83	3.04 ± 0.90	0.877
HDL-c (mmol/L)	1.03 ± 0.28	1.09 ± 0.29	0.177
TG (mmol/L)	1.28 (0.97–1.97)	1.44 (1.10–2.04)	0.246
ALB (g/L)	39.0 ± 4.3	38.4 ± 3.8	0.370
P (mmol/L)	1.27 ± 0.22	1.23 ± 0.19	0.158
Na (mmol/L)	140.3 ± 3.2	140.5 ± 2.6	0.670
Cl (mmol/L)	103.5 ± 3.7	103.6 ± 3.0	0.869
UREA (mmol/L)	5.0 (4.2–6.0)	4.9 (4.2–6.1)	0.918
UA (*μ*mol/L)	349.5 ± 99.9	337.6 ± 96.1	0.400
eGFR (mL·min^−1^·1.73 m^−2^)	102.0 ± 27.9	97.2 ± 33.9	0.309
UACR (mg/g)	6.2 (4.4–10.6)	11.5 (6.0–21.2)	<0.001

*Echocardiography*			
EF (%)	67.7 ± 5.6	67.1 ± 7.8	0.602
AO (mm)	25.5 ± 3.3	25.9 ± 3.7	0.401
LA (mm)	29.9 ± 3.9	32.1 ± 3.9	<0.001
E/A	^a^(253) 0.82 (0.72–1.21)	(61) 0.70 (0.65–0.81)	<0.001

*LV geometry*			
Normal, *n* (%)	72 (22.7)	—	—
Concentric remodeling, *n* (%)	183 (57.7)	—	—
Eccentric hypertrophy, *n* (%)	—	12 (3.8)	—
Concentric hypertrophy, *n* (%)	—	50 (15.8)	—

SBP = systolic blood pressure; DBP = diastolic blood pressure; ACEI/ARB: angiotensin-converting enzyme inhibitor/angiotensin receptor blocker; CCB: calcium channel blocker; HbA1c = hemoglobin A1c; HOMA-IR = homeostasis model assessment ratio; EF = ejection fraction; AO = aortic root; LA = left atrial. ^a^The remaining valid data regardless of the missing ones.

**Table 2:** Characteristics of subjects categorized by UACR quartiles.

Variables	UACR (mg/g)				*P* value
	0–4.4 *n* = 102	4.4–7.1 *n* = 56	7.1–12.4 *n* = 80	12.4–30 *n* = 79	
Age (years)	52.5 ± 10.5	53.8 ± 10.0	56.5 ± 13.2	58.2 ± 13.7	0.009
Female, *n* (%)	23 (22.5)	23 (41.1)	39 (48.8)	40 (50.6)	<0.001
Smoking, *n* (%)	34 (33.3)	13 (23.2)	15 (18.8)	15 (19.0)	0.068
Hypertension, *n* (%)	28 (27.5)	17 (30.4)	27 (33.8)	47 (59.5)	<0.001
Carotid plaque, *n* (%)	13 (12.7)	9 (16.1)	14 (17.5)	14 (17.7)	0.774
Duration of diabetes (years)	5 (1–9)	7 (1–10)	6 (1–10)	6 (1–12)	0.419
SBP (mmHg)	129.4 ± 15.5	134.4 ± 15.5	133.2 ± 18.0	139.5 ± 21.0	0.003
DBP (mmHg)	81.3 ± 9.2	79.7 ± 10.7	80.6 ± 10.3	82.1 ± 12.9	0.899
BMI (kg/m^2^)	24.7 ± 3.4	24.8 ± 4.0	24.4 ± 4.3	24.5 ± 4.2	0.923
Obesity, *n* (%)	12 (11.8)	11 (19.6)	14 (17.5)	10 (12.7)	0.469

*Medication history*					
ACEI/ARB use, *n* (%)	15 (14.7)	10 (17.9)	17 (21.3)	27 (34.2)	0.014
Statin use, *n* (%)	45 (44.1)	24 (42.9)	27 (33.8)	36 (45.6)	0.416
CCB use, *n* (%)	11 (10.8)	8 (14.3)	8 (10.3)	14 (17.7)	0.435
*β*-Blocker, *n* (%)	5 (4.9)	5 (8.9)	4 (5.0)	2 (2.5)	0.422

*Laboratory values*					
HbA1c (%)	7.6 (6.4–9.8)	7.9 (6.2–10.1)	9.7 (7.8–11.1)	9.2 (7.2–10.8)	<0.001
HOMA-IR	1.65 (0.71–3.08)	1.24 (0.70–2.90)	1.50 (0.74–3.83)	1.35 (0.60–2.46)	0.643
FPG (mmol/L)	6.6 (5.3–8.3)	6.9 (4.9–8.7)	7.8 (6.0–9.9)	6.5 (5.2–8.5)	0.026
Anemia, *n* (%)	7 (6.9)	3 (5.4)	6 (7.5)	13 (16.5)	0.074
CHOL (mmol/L)	4.83 ± 1.03	4.96 ± 1.20	4.74 ± 0.99	4.75 ± 1.06	0.638
LDL-c (mmol/L)	3.07 ± 0.82	3.13 ± 1.03	3.02 ± 0.73	2.92 ± 0.82	0.492
HDL-c (mmol/L)	1.04 ± 0.29	1.01 ± 0.22	1.06 ± 0.31	1.06 ± 0.27	0.699
TG (mmol/L)	1.26 (0.95–1.95)	1.33 (1.04–2.13)	1.28 (0.93–1.80)	1.44 (1.09–2.03)	0.445
ALB (g/L)	40.3 ± 3.3	39.3 ± 3.3	37.9 ± 4.8	37.7 ± 4.6	<0.001
P (mmol/L)	1.26 ± 0.23	1.29 ± 0.21	1.26 ± 0.20	1.26 ± 0.23	0.882
Na (mmol/L)	140.9 ± 2.4	140.4 ± 3.5	140.5 ± 2.4	139.6 ± 4.0	0.054
Cl (mmol/L)	104.1 ± 3.2	103.8 ± 3.7	103.2 ± 2.9	103.1 ± 4.4	0.201
UREA (mmol/L)	5.1 (4.5–6.0)	5.5 (4.2–6.2)	5.0 (4.0–6.0)	4.8 (3.9–5.7)	0.550
UA (*μ*mol/L)	367.4 ± 91.5	347.6 ± 98.3	334.7 ± 96.9	333.2 ± 108.6	0.068
eGFR (mL·min^−1^·1.73 m^−2^)	96.0 ± 20.4	100.6 ± 27.4	107.5 ± 31.6	101.2 ± 36.0	0.164

*Echocardiography*					
EF (%)	68.4 (64.0–72.0)	69.9 (64.2–72.1)	67.0 (64.7–72.7)	67.0 (64.0–71.0)	0.510
AO (mm)	25.9 ± 3.0	25.1 ± 4.0	25.9 ± 3.5	25.1 ± 3.4	0.216
LA (mm)	33.0 ± 3.6	30.5 ± 4.1	29.8 ± 3.8	31.1 ± 4.6	0.193
LVIDd (mm)	42.7 ± 3.9	42.7 ± 4.6	41.5 ± 4.4	41.5 ± 4.4	0.108
LVIDs (mm)	26.5 ± 3.2	26.2 ± 3.0	25.9 ± 3.7	26.2 ± 3.9	0.777
IVSd (mm)	10.5 ± 1.3	10.6 ± 1.6	10.5 ± 1.7	11.3 ± 1.8	0.008
LVPWd (mm)	9.8 ± 1.3	9.9 ± 1.5	9.9 ± 1.4	10.5 ± 1.6	0.003
RWT	0.46 ± 0.07	0.47 ± 0.10	0.48 ± 0.09	0.51 ± 0.10	0.001
LVMI (g/m^2,7^)	36.4 ± 8.0	38.3 ± 8.7	37.2 ± 8.1	41.7 ± 11.5	0.003
E/A	0.82 (0.74–1.19)	0.81 (0.73–1.16)	^a^(79) 0.80 (0.67–1.14)	(77) 0.73 (0.65–0.88)	0.008
LVH, *n* (%)	11 (10.8)	9 (16.1)	13 (16.3)	29 (36.7)	<0.001

LVIDd = left ventricular internal dimension diastole; LVIDs = left ventricular internal systole; IVSd = interventricular septal dimension; LVPWd = left ventricular posterior wall dimension. ^a^The remaining valid data regardless of the missing ones.

**Table 3:** Univariate and multiple linear regression analysis to include log_2_ UACR for other clinical parameters.

Explanatory variable	Univariate regression			Multiple regression		
	Regression coefficient	Standard error	*P* value	Regression coefficient	Standard error	*P* value
HbA1c (%)	0.085	0.022	<0.001	0.090	0.021	<0.001
Hypertension	0.527	0.107	<0.001	0.498	0.131	<0.001
ALB (g/L)	−0.052	0.012	<0.001	−0.037	0.012	0.003
Age (years)	0.018	0.004	<0.001	0.005	0.004	0.251
Gender (female)	0.145	0.032	<0.001	0.238	0.116	0.041
Smoking	−0.331	0.124	0.008	−0.211	0.127	0.097
ACEI/ARB use	0.433	0.128	0.001	0.040	0.149	0.789

**Table 4:** Multinomial logistic regression analysis to include log_2_ UACR for LVH.

	*B*	SE	Wald	df	*P*	OR	95% CI
Gender (female)	1.373	0.359	14.591	1	<0.001	3.946	1.951–7.981
Age (years)	0.058	0.016	13.506	1	<0.001	1.059	1.027–1.093
Hypertension	1.174	0.355	10.963	1	0.001	3.236	1.615–6.486
Obesity	1.742	0.437	15.869	1	<0.001	5.708	2.423–13.447
Log_2_ UACR (mg/g)	0.417	0.185	5.064	1	0.024	1.517	1.055–2.182

**Table 5:** OR (95% CI) of left ventricular hypertrophy according to UACR.

	UACR (mg/g)		Continuous variables log_2_ UACR (mg/g)
	0–10.2	10.2–30.0	
*N*	255	62	317
Unadjusted	1.000 (reference)	4.725 (2.635–8.475), <0.001	2.023 (1.493–2.740), <0.001
Model 1	1.000 (reference)	3.413 (1.753–6.644), <0.001	1.663 (1.161–2.382), 0.006
Model 2	1.000 (reference)	3.162 (1.593–6.274), 0.001	1.529 (1.062–2.202), 0.022
Model 3	1.000 (reference)	3.104 (1.557–6.188), 0.001	1.511 (1.047–2.180), 0.028

Values are OR (95% CI) and *P* value; model 1: adjusted for age, gender, and obesity; model 2: further adjusted for hypertension, carotid plaque, and duration of diabetes; model 3: further adjusted for smoking, the use of ACEI or ARB, and statin medication.

## Data Availability

The data used to support the findings of this study are available from the corresponding author upon request.

## References

[1] Lovic D., Erdine S., Burak Catakoglu A. (2014). How to estimate left ventricular hypertrophy in hypertensive patients. *Anadolu Kardiyoloji Dergisi/The Anatolian Journal of Cardiology*.

[2] Galderisi M., Anderson K. M., Wilson P. W. F., Levy D. (1991). Echocardiographic evidence for the existence of a distinct diabetic cardiomyopathy (the Framingham Heart Study). *The American Journal of Cardiology*.

[3] Deng T., Ou B., Zhu T., Xu D. (2019). The effect of hypertension on cardiac structure and function in different types of hypertrophic cardiomyopathy: a single-center retrospective study. *Clinical and Experimental Hypertension*.

[4] Dawson A., Morris A. D., Struthers A. D. (2005). The epidemiology of left ventricular hypertrophy in type 2 diabetes mellitus. *Diabetologia*.

[5] Liao Y., Cooper R. S., Mcgee D. L., Mensah G. A., Ghali J. K. (1995). The relative effects of left ventricular hypertrophy, coronary artery disease, and ventricular dysfunction on survival among black adults. *JAMA: The Journal of the American Medical Association*.

[6] Cooper R. S., Simmons B. E., Castaner A., Santhanam V., Ghali J., Mar M. (1990). Left ventricular hypertrophy is associated with worse survival independent of ventricular function and number of coronary arteries severely narrowed. *The American Journal of Cardiology*.

[7] Artham S. M., Lavie C. J., Milani R. V., Patel D. A., Verma A., Ventura H. O. (2009). Clinical impact of left ventricular hypertrophy and implications for regression. *Progress in Cardiovascular Diseases*.

[8] Chinese Society of Diabetology of Chinese Medical Association (2018). Guideline for the prevention and control of type 2 diabetes in China (2017 edition). *Chinese Journal of Practical Internal Medicine*.

[9] Deckert T., Feldt-Rasmussen B., Borch-Johnsen K., Jensen T., Kofoed-Enevoldsen A. (1989). Albuminuria reflects widespread vascular damage. *Diabetologia*.

[10] Smith E. R., Zurakowski D., Saad A., Scott R. M., Moses M. A. (2008). Urinary biomarkers predict brain tumor presence and response to therapy. *Clinical Cancer Research*.

[11] Cho S. H., Oh Y. J., Nam A. (2007). Evaluation of serum and urinary angiogenic factors in patients with endometriosis. *American Journal of Reproductive Immunology*.

[12] Hou L. N., Li F., Zeng Q. C. (2014). Excretion of urinary orosomucoid 1 protein is elevated in patients with chronic heart failure. *PLoS One*.

[13] American Diabetes Association (2019). Standards of medical care in diabetes-2019 abridged for primary care providers. *Clinical Diabetes*.

[14] Sung K. C., Ryu S., Lee J. Y. (2016). Urine albumin/creatinine ratio below 30 mg/g is a predictor of incident hypertension and cardiovascular mortality. *Journal of the American Heart Association*.

[15] Schiller N. B., Shah P. M., Crawford M. (1989). Recommendations for quantitation of the left ventricle by two-dimensional echocardiography. *Journal of the American Society of Echocardiography*.

[16] Chinese Society of Health Management of Chinese Medical Association (2015). Standard for carotid ultrasound examination in Chinese healthy population. *Chinese Journal of Health Management*.

[17] Group of Anemia and Erythrocyte (2018). Multidisciplinary expert consensus for the diagnosis, treatment and prevention of iron deficiency and iron-deficiency anemia. *National Medical Journal of China*.

[18] Wang Y., Mi J., Shan X.-Y., Wang Q. J., Ge K.-Y. (2007). Is China facing an obesity epidemic and the consequences? The trends in obesity and chronic disease in China. *International Journal of Obesity*.

[19] Levey A. S. (2000). A simplified equation to predict glomerular filtration rate from serum creatinine. *Journal of the American Society of Nephrology*.

[20] Matthews D. R., Hosker J. P., Rudenski A. S., Naylor B. A., Treacher D. F., Turner R. C. (1985). Homeostasis model assessment: insulin resistance and *β*-cell function from fasting plasma glucose and insulin concentrations in man. *Diabetologia*.

[21] Marwick T. H., Gillebert T. C., Aurigemma G. (2015). Recommendations on the use of echocardiography in adult hypertension: a report from the European Association of Cardiovascular Imaging (EACVI) and the American Society of Echocardiography (ASE). *European Heart Journal -Cardiovascular Imaging*.

[22] Diercks G., Boven A. J. V., Hillege H. L. (2000). Microalbuminuria is independently associated with ischaemic electrocardiographic abnormalities in a large non-diabetic population. The PREVEND (Prevention of REnal and Vascular ENdstage Disease) study. *European Heart Journal*.

[23] Kramer H., Jacobs D. R., Bild D. (2005). Urine albumin excretion and subclinical cardiovascular disease. *Hypertension*.

[24] Wachtell K., Ibsen H., Olsen M. H. (2003). Albuminuria and cardiovascular risk in hypertensive patients with left ventricular hypertrophy: the LIFE study. *Annals of Internal Medicine*.

[25] Gutiérrez O. M., Khodneva Y. A., Paul M. (2013). Association between urinary albumin excretion and coronary heart disease in black vs white adults. *Journal of the American Medical Association*.

[26] Siddique A., Murphy T. P., Naeem S. S. (2019). Relationship of mildly increased albuminuria and coronary artery revascularization outcomes in patients with diabetes. *Catheterization and Cardiovascular Interventions*.

[27] Maiello M., Zito A., Carbonara S., Ciccone M. M., Palmiero P. (2017). Left ventricular mass, geometry and function in diabetic patients affected by coronary artery disease. *Journal of Diabetes and its Complications*.

[28] Witham M. D., Davies J. I., Dawson A., Davey P. G., Struthers A. D. (2004). Hypothetical economic analysis of screening for left ventricular hypertrophy in high-risk normotensive populations. *Quarterly Journal of Medicine*.

[29] Devereux R. B., Pickering T. G., Harshfield G. A. (1983). Left ventricular hypertrophy in patients with hypertension: importance of blood pressure response to regularly recurring stress. *Circulation*.

[30] Lorenzo-Almorós A., Tuñón J., Orejas M., Cortés M., Egido J., Lorenzo Ó (2017). Diagnostic approaches for diabetic cardiomyopathy. *Cardiovascular Diabetology*.

[31] Pham I., Cosson E., Nguyen M. T. (2015). Evidence for a specific diabetic cardiomyopathy: an observational retrospective echocardiographic study in 656 asymptomatic type 2 diabetic patients. *Insternational Journal of Endocrinology*.

[32] Somaratne J. B., Whalley G. A., Poppe K. K. (2011). Screening for left ventricular hypertrophy in patients with type 2 diabetes mellitus in the community. *Cardiovascular Diabetology*.

[33] Palmiero P., Zito A., Maiello M. (2015). Left ventricular diastolic function in hypertension: methodological considerations and clinical implications. *Journal of Clinical Medicine Research*.

[34] Miyashita K., Itoh H., Tsujimoto H. (2009). Natriuretic peptides/cGMP/cGMP-dependent protein kinase cascades promote muscle mitochondrial biogenesis and prevent obesity. *Diabetes*.

[35] Peter A. (2005). Human fat cell lipolysis: biochemistry, regulation and clinical role. *Best Practice & Research Clinical Endocrinology & Metabolism*.

[36] Liu Z., Tian H., Hua J. (2019). A CRM1 inhibitor alleviates cardiac hypertrophy and increases the nuclear distribution of NT-PGC-1*α* in NRVMs. *Frontiers in Pharmacology*.

[37] Naresh C. N., Hayen A., Weening A., Craig J. C., Chadban S. J. (2013). Day-to-day variability in spot urine albumin-creatinine ratio. *American Journal of Kidney Diseases*.

[38] Zelmanovitz T., Gross J. L., Oliveira J. R., Paggi A., Tatsch M., Azevedo M. J. (1997). The receiver operating characteristics curve in the evaluation of a random urine specimen as a screening test for diabetic nephropathy. *Diabetes Care*.

